# The Role of MicroRNA 181d as a Possible Biomarker Associated With Tumor Progression in Meningiomas

**DOI:** 10.7759/cureus.19158

**Published:** 2021-10-31

**Authors:** Vinícius Carneiro, Múcio Cirino, Rodrigo Panepucci, Fernanda Peria, Daniela Tirapelli, Benedicto Colli, Carlos Gilberto Carlotti Jr

**Affiliations:** 1 Surgery and Anatomy, University of São Paulo, Ribeirão Preto Medical School, Ribeirão Preto, BRA; 2 Hemocenter, Laboratory of Functional Biology (LFBio) Center for Cell-Based (CTC, Regional Blood Center of Ribeirão Preto, Ribeirão Preto, BRA; 3 Neurosurgery, University of São Paulo, Ribeirão Preto Medical School, Ribeirão Preto, BRA

**Keywords:** biomarker, tumor progression, mir-181d, micrornas, meningiomas

## Abstract

Introduction

Meningiomas are slow-growing intracranial neoplasms that originate from arachnoid meningothelial cells and represent 13-26% of intracranial tumors, thus being the most common. There are numerous technological advances available for a better understanding of the molecular pathways correlated with tumorigenesis and tumor progression of meningiomas. In this context, the role of microRNAs (miRNAs), which are non-coding RNAs (ncRNAs) consisting of 18 to 25 nucleotides whose function is the silencing of mRNA at the posttranscriptional level, has been highlighted. Recent studies suggest that miRNAs may act as possible biomarkers as well as therapeutic targets for various diseases, including brain tumors. Therefore, the objective of our study was to evaluate the tissue and plasma expression of the miRNAs miR-181d, miR-181c, and miR-130a.

Methods

The miRNAs miR-181d, miR-181c, and miR-130a were selected from our group’s prior study by the large-scale microarray analysis technique. In this work, the expression of these miRNAs in the tumor tissue and plasma of patients with grade I (16 patients), II (16 patients), and III (eight patients) meningiomas was evaluated.

Results

MiR-181d was overexpressed in both tumor tissue and plasma in the studied groups. The level of expression was higher according to the progression of tumor grade. MiR-181c and miR-130a showed no significant difference in the studied groups in either tumor tissue or plasma.

Conclusions

MiR-181d has potential as a biomarker for meningiomas and is associated with the tumor progression of meningiomas.

## Introduction

Meningiomas have an annual incidence of approximately six out of every 100,000 people, making them the most common intracranial tumors and affecting mainly adults, especially women, and after the fifth decade of life.

When benign, these tumors are susceptible to surgical healing, and they should have surgery as the first treatment option. The emergence of microsurgical techniques and a better anatomical understanding of these tumors has led to reduced morbidity and mortality, as well as longer survival. However, in some cases, either because of its location in intricate areas or at the base of the skull, complete surgical removal cannot be achieved. In the last 20 years, many studies aiming for a better biological understanding of meningiomas have been carried out in search of new treatments. Thus, a more accurate understanding of biological pathways and their natural history will allow us in the future to have therapeutic alternatives to achieve a cure for meningiomas [1-5].

According to the World Health Organization (WHO) classification, meningiomas are divided into three types: benign (WHO grade I), atypical (WHO grade II), and anaplastic (WHO grade III) [3,6,7]. Different histological parameters are used for grading benign, atypical, and anaplastic lesions. The mitotic index is considered the most important factor in determining whether meningioma is atypical and/or anaplastic. In addition, cellularity is also important; the presence of small cells with a high nucleus-cytoplasm relationship and prominent nucleoli and geographic pattern necrosis are also important. MIB 3, the Ki-67 monoclonal antigen, has been used in several meningioma studies as an additional support in its grading. Thus, high MIB 1 levels have been associated with aggressiveness, poor prognosis, and/or residual tumor growth [8]

The WHO classification was based on morphological parameters, and the last edition (fourth) opened the door to a molecular era. However, although studies have shown great progress in understanding the molecular mechanisms to elucidate tumorigenesis and meningioma progression, in the 4th edition, no changes were suggested in the classification of meningiomas. Analysis of chromosomal regions and studies of new genes should assist in diagnosis and prognosis in the future, and possible molecular markers may eventually facilitate histological and molecular combinations in the classification of meningiomas. Using global genome analysis, such as microarray techniques and gene expression analysis, will contribute to and accelerate the process of developing new therapies. However, little is known about the main pathways correlated with these neoplasms [6-9].

Meningiomas are tumors that present well-characterized cytogenetic alterations. Deletion of the long arm (q) of chromosome 22 is the most frequent, present in up to 70% of sporadic meningiomas, and correlated with tumor initiation [6-10]. Deletion of the short arm (p) of chromosome one is the second most common. The presence in 70% of atypical and almost 100% of anaplastic tumors indicates a correlation with tumor progression [11]. Recently, a new class of non-coding RNAs, microRNAs, was discovered. These molecules act as potent posttranscriptional regulators of gene expression. Studies emphasize the importance of these molecules in reporting changes in microRNA expression in different diseases. MicroRNAs are stable in plasma, and their expression profile changes under different physiological and pathological conditions; thus, they can be used as potential biomarkers [12].

MicroRNAs are expressed at different levels and specifically in different tissues. Bioinformatics tools predict thousands of mRNA targets for each miRNA, suggesting that many genes are subject to miRNA-mediated regulation [13]. Therefore, miRNAs can be used as a tool for diagnosis and prognosis. In addition, new therapeutic strategies involving silencing miRNAs or mimicking miRNAs could be proposed based on the functions of these small non-coding RNAs as oncogenes and tumor suppressor genes in brain tumors [14].

The overall expression profile of microRNAs in meningiomas was analyzed using the microarray technique, and three of these microRNAs were different expressed in this global analysis (miR-130a, miR-181c, and miR-181d). Then they were selected as differentially expressed molecular candidates. Therefore, the aim of this study was to validate the expression of miR-130a, miR-181c, and miR-181d, as well as to analyze their possible role as biomarkers and their correlation with the tumor progression of meningiomas.

## Materials and methods

Tumor tissue and plasma samples were collected from 40 patients with confirmed histological diagnosis of meningioma according to the WHO criteria.

Three groups were defined consisting of grade I meningiomas (16 patients), grade II meningiomas (16 patients), and grade III meningiomas (eight patients).

As a control group, six arachnoid samples were obtained from patients undergoing cerebral aneurysm surgery.

All patients involved in the research were approached preoperatively, and each freely consented by signing the informed consent form. This study was approved by the ethics committee of the Clinic Hospital of Ribeirão Preto.

RNA isolation and real-time polymerase chain reaction

Total RNA was extracted with TRIzol reagent (Applied Biosystems, Waltham, Massachusetts) and an RNeasy Mini Kit (QIAGEN, Hilden, Germany) according to the manufacturer’s instructions. In preparation for real-time polymerase chain reaction (PCR), reverse transcription of RNA samples was performed using the High-Capacity cDNA kit (Applied Biosystems).

Real-time PCR

The complementary DNA (cDNA) was amplified with quantitative real-time polymerase chain reaction (q-PCR) using TaqMan Master Mix (Applied Biosystems) for the reaction of microRNAs and gene expression. The U6 gene was used as an endogenous control (housekeeping) for the reaction of the microRNAs; however, for the gene reaction, TBP and HPRT were used as endogenous controls. The PCR conditions were as follows: preheating at 50°C for two min, denaturation at 95°C for 10 min and 50 cycles of amplification and quantification (15 sec at 95°C and one min at 60°C). All reactions were carried out in duplicate and analyzed with the 7500 Sequence Detection System apparatus (Applied Biosystems). The data were analyzed using ABI-7500 SDS software. Dissociation curves were generated (melting curves) after amplification by RQ-PCR. The samples that showed dissociation curves with different temperatures and/or more than one point of dissociation in the same sample were discarded and repeated.

Statistical analysis

The statistics and data analysis were performed using ABI Prism and GraphPad Prism 5 software.

Data availability

The data associated with the paper are available in the Central Library USP Ribeirão Preto repository; they are not publicly available but are available from the corresponding author on reasonable request. Such data will not be published as supplementary digital material.

## Results

**Figure 1:**
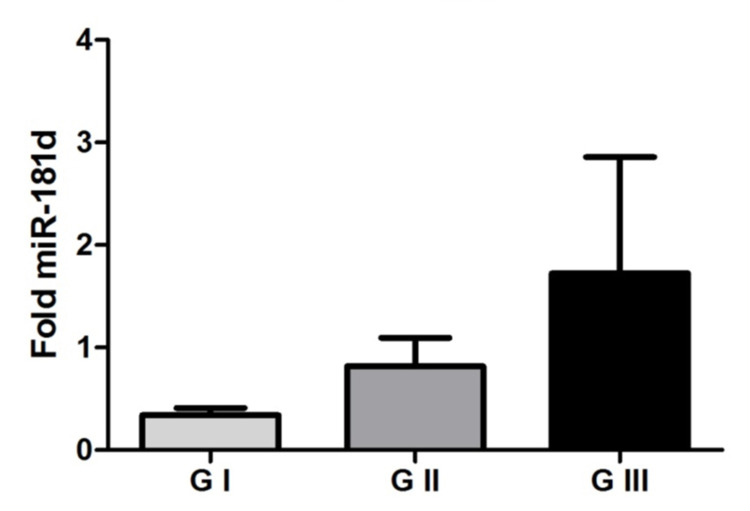
MicroRNA-181d in tumor tissue Representation of mean values (± standard error) of microRNA-181d expression among the studied groups. There was a significant difference between the groups of grade I meningioma, grade II meningioma, and grade III meningioma (p = 0.0207, Kruskal Wallis test).

**Figure 2:**
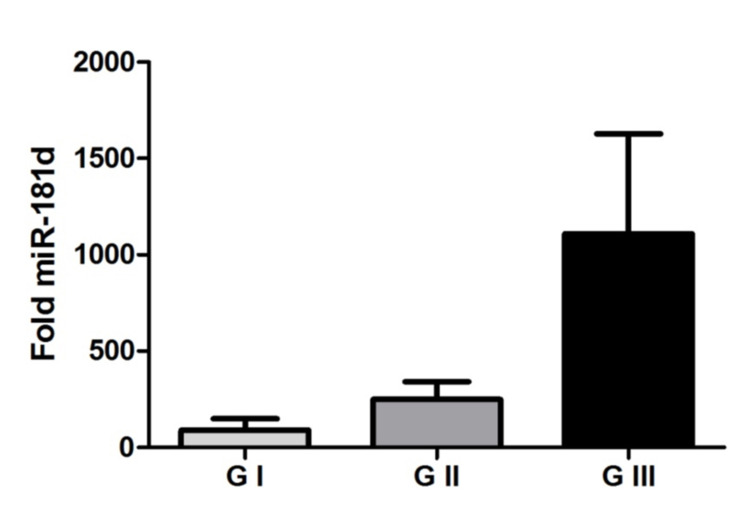
MicroRNA-181d in the plasma Representation of mean values (± standard error) of microRNA-181d expression among the studied groups. There was a significant difference between the groups of grade I meningioma, grade II meningioma, and grade III meningioma (p = 0.0187, Kruskal Wallis test).

Our results demonstrated an increase in miR-181d expression in meningiomas compared to the arachnoid control group. This increase in miR-181d expression was even greater according to the progression of the tumor grade, as shown in Figure 1. Another relevant finding was that in plasma the expression of miR-181d was even more evident and followed the progression of the tumor grade as well (Figure 2). Grade III meningioma has double the spread compared to grade I meningioma. There was statistical significance in tissue and plasma evaluation.

**Figure 3:**
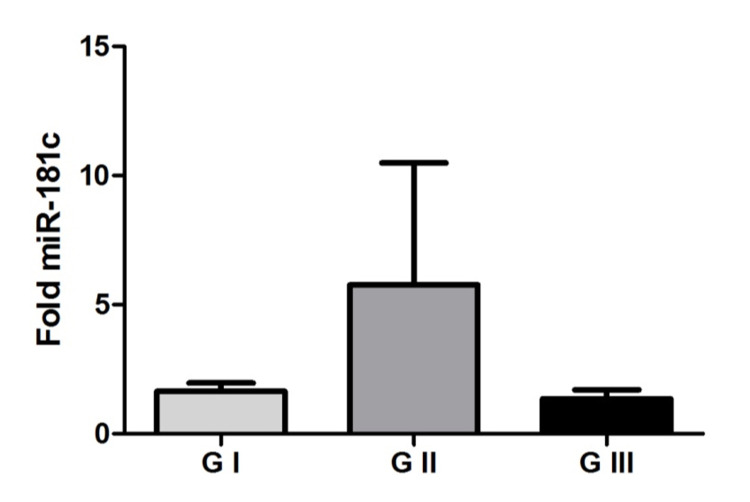
MicroRNA-181c in tumor tissue Representation of mean values (± standard error) of microRNA-181c expression among the studied groups. There was no significant difference between the grade I meningioma, grade II meningioma, and grade III meningioma groups (p = 0.8236 Kruskal Wallis test).

**Figure 4:**
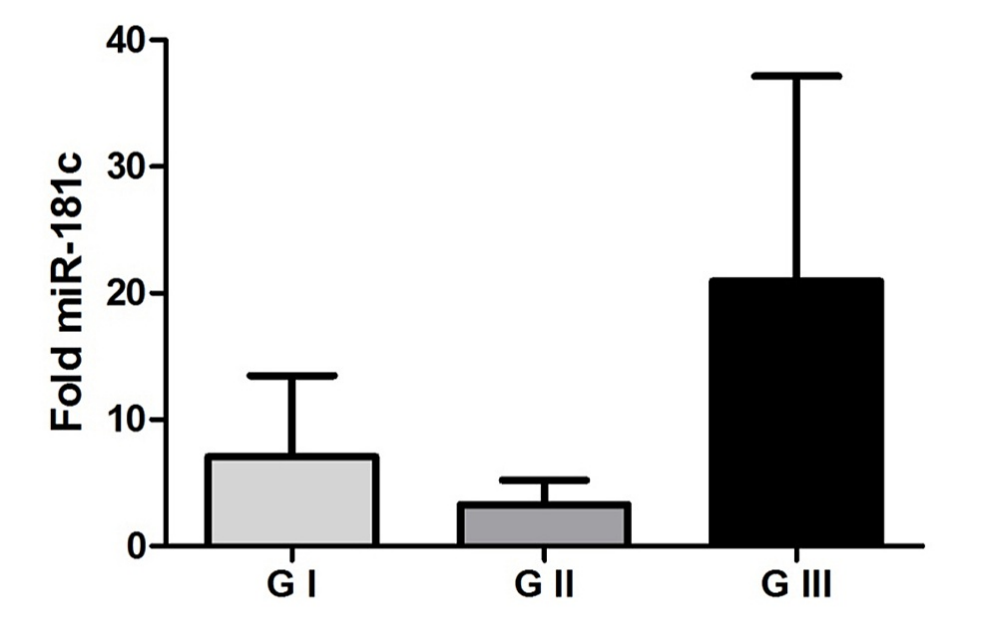
MicroRNA-181c in the plasma Representation of mean values (± standard error) of microRNA-181c expression among the studied groups. There was no significant difference between the grade I meningioma, grade II meningioma, and grade III meningioma groups (p = 0.5603 Kruskal Wallis test).

Our results did not demonstrate statistical significance in miR-181c expression between the studied groups in either tumor tissue (p = 0.8236 Kruskal Wallis test) or plasma (p = 0.5603 Kruskal Wallis test), as can be seen in Figure 3 and 4. Thus, it cannot be said whether there is a difference in the expression of this microRNA in meningiomas.

**Figure 5:**
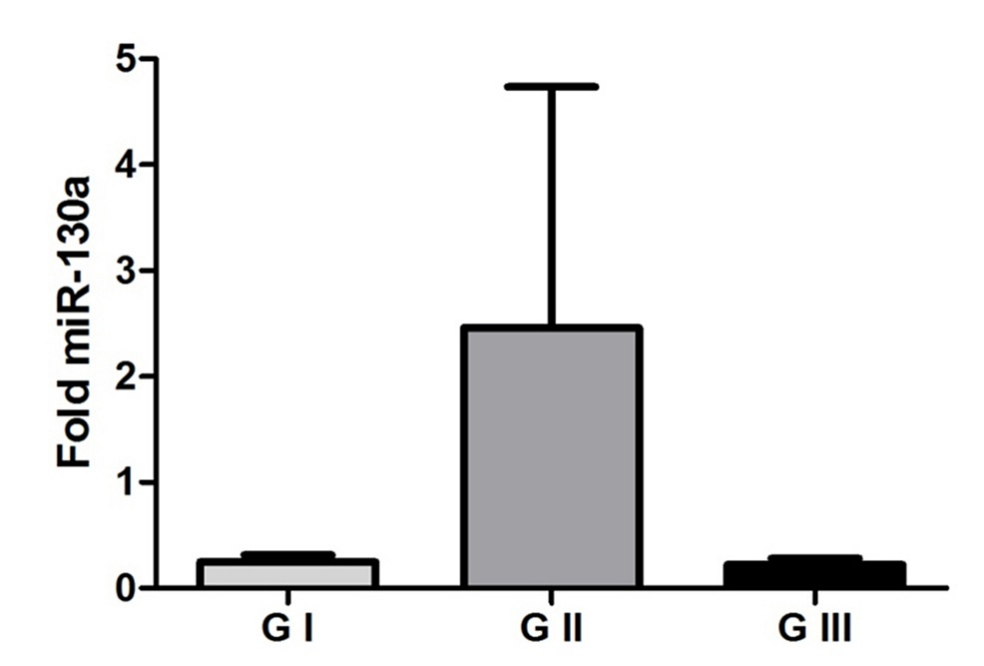
MicroRNA-130a in tumor tissue Representation of mean values (± standard error) of microRNA-130a expression among the studied groups. There was no significant difference between the grade I meningioma, grade II meningioma, and grade III meningioma groups (p = 0.9179, Kruskal Wallis test).

There was no statistical significance (p = 0.9179, Kruskal Wallis test) in the expression of miR-130a between the groups studied in any tumor tissue (Figure 5). And the plasma samples of this microRNA were not amplified.

## Discussion

The first two studies that evaluated the profile of miRNA expression in glioblastoma multiform (GBM) were published in 2005 [15, 16]. Ciafre et al. [15] observed the global expression of 245 miRNAs in GBM using the microarray technique, which allowed us to identify their altered expression. Several miRNAs, including miR-21, were highly expressed in these tumors. Chan et al. [15] were the first to investigate the functional properties of a miRNA and found that inhibition of miRNA-21 resulted in a significant increase in apoptosis. Thus, they concluded that miR-21 could function as an oncogene. Since then, other microRNAs have been described as altered in GBM and may act as tumor suppressors or oncogenes. However, few studies have evaluated the profile of miRNA expression in meningiomas. Zhi et al. [17] identified 14 miRNAs with different expression profiles in meningiomas; among them, miR-190a high expression and mi-R29c-3p and miR-219-5p low expression correlated with high recurrence rates. These results suggest their use as biomarkers in the diagnosis and prognosis of meningiomas. Saydam et al. [18] demonstrated that low levels of miR-200a contribute to meningioma tumorigenesis by increasing the expression of three different mRNAs. These miR-200a-mediated processes may be present in other tumors and provide potential targets for therapeutic intervention.

Recent studies have demonstrated the role of microRNAs from the miR-181 family in gliomagenesis pathways, as well as the involvement of these miRNAs in glioma resistance to radiotherapy and chemotherapy treatments [19,20]. However, there are no reports of the role of the 181 family of miRNAs in meningiomas.

Zhang et al. [21] analyzed miR-181d expression in 82 cases of glioblastomas and observed that miR-181d expression was associated with longer survival. The regulation of the MGMT gene by miR-181d was found after transfection of this miRNA in glioblastoma cell lines, as the transfection caused a reduction in the expression of the MGMT gene as well as the MGMT protein. Conversely, low expression levels of miR-181d were associated with increased expression of MGMT.

Our results demonstrated an increase in miR-181d expression in meningiomas, and we also observed that the increase in miR-181d expression was even higher according to tumor grade progression. Another relevant finding was that in the plasma, the expression of miR-181d was even more evident and accompanied the progression of tumor grade. From these results, we can suggest a possible role of miR-181d in the progression of meningiomas, as well as its potential to have an important biomarker function.

In recent years, many studies have demonstrated the role of microRNAs as biomarkers due to their detection and greater stability than mRNAs, making them less susceptible to chemical modification and degradation. Another important feature is the regulation of a wide range of targets by miRNAs, which can be explored in the diagnosis, prognosis, and search for new therapeutic targets [22]. In meningiomas, microRNA biomarkers in serum could be developed as a convenient and effective indicator to diagnose and monitor meningioma’s; our results demonstrate that miR-181d is a promising candidate. Little studies have demonstrated a plasmatic profile of microRNA expression in patients with meningiomas, evidencing eventual usefulness for tracking and monitoring these patients [23,24]

Lakomy et al. [25] analyzed 38 samples of patients diagnosed with glioblastomas who presented 32% methylation of MGMT by real-time PCR. High expression of miR-21 and miR-181c was also observed, and this high expression was associated with early progression. The combination of increased expression of these two miRNAs predicts greater efficacy of progression. The authors suggest that the combination of miR-181c and miR-21 expression levels have high sensitivity and specificity as an indicator of early progression. Slaby et al. [19] demonstrated reduced expression of miRNA-181b and 181c in patient samples that responded to combined radiotherapy and chemotherapy.

Our results showed no difference in miR-181c expression between the groups studied in either tumor tissue or plasma samples. There is no involvement of the all miR-181 family but only a specificity of miR-181d in meningiomas.

In addition to miR-181d and miR-181c, miR-130a was also selected in the initial microarray analyses. miR-130a also stands out in the literature and is associated with various types of tumors.

Ouyang et al. [26] analyzed 15 cases of breast cancer using the microarray technique and used adjacent normal breast tissue as a control group. A total of 1513 miRNAs were studied, and the authors also selected 11 genes based on bioinformatics analysis (starBase platform) and literature review. Five of these miRNAs showed high expression levels (miR-155-5p, miR-21-3p, miR-181a-5p, miR-181b-5p and miR-183-5p), and six miRNAs were downregulated (miR10b-5p, miR451a, miR125b-5p, miR31-5p, miR195-5p and miR130a-3p). The genes were validated by real-time PCR, and the main pathways were phosphatase and tensin homolog (PTEN)/ protein kinase B (Akt), mitogen-activated protein kinase (MAPK), multidrug resistance protein 1 (MDR1), Ras homolog family member A (RhoA), forkhead box O3 (FOXO3), and programmed cell death 4 (PDCD4). In the same study, in vitro experiments of miRNA-130a demonstrated an important role as an oncomiR since one of its targets is MAPK, which can promote cell proliferation and angiogenesis. In these experiments, the expression of miRNAs miR130-a and miR-451a significantly changed when subjected to doxorubicin treatment, so the authors concluded that altered expression of these miRNAs is mainly related to chemoresistance.

He et al. [27], in an in vitro study with a cervical cancer cell line, demonstrated that miR-130a directly regulates Dicer enzyme mRNA and increases cell migration and invasion. High miR-130a expression is significantly associated with poor survival. Thus, miR130a-regulated expression of Dicer is an important potential prognostic factor in cervical cancer. The immunohistochemical technique evaluated the protein expression of Dicer as 51% positivity, suggesting a posttranscriptional control mechanism. Dicer mRNA and protein expression are associated with distant metastases and recurrence.

In our study, miR-130a showed no difference in the grade I, grade II, and grade III meningioma groups, which may suggest a specificity of this miRNA for some tumors.

Qiu et al. [28] analyzed the expression of miRNAs and genes in 480 glioblastoma samples (318 cases with relapse and 162 cases without progression and/or recurrence) and compared them with 10 samples of normal brain tissue. There were 534 miRNAs with altered expression levels and 20 miRNAs correlated with clinical data. Six of these miRNAs were selected for RT-PCR validation: miR-326 and miR-130a were hyper expressed, and miR-323, miR-329, miR-155, and miR210 were hypo expressed with a significant association with longer survival. High expression levels of miR-326 and miR-130a miRNAs and low expression levels of miR-155 and miR-210 miRNAs were related to longer progression-free time. The authors concluded that miRNAs miR326, miR130a, miR155, and miR210 and their interactions may serve as prognostic factors and survival markers in GBM.

## Conclusions

From our results and according to information in the literature, we envision an important role for miRNAs in the formation and progression of brain tumors. Specifically, in meningiomas, miR181d has a likely role in tumor progression, as it is elevated in samples compared to arachnoid, and this increase is even greater with tumor progression. Findings were similar in plasma, with greater intensity, evidencing an important role as a biomarker. There is a possibility of having another tool for monitoring these patients, allowing the detection of both relapse and an eventual malignancy through plasma evaluation.

The identification of this specific microRNA alteration in meningioma is important for a better molecular understanding. The next step will be a characterization of which molecular pathway is being controlled by miR-181d through studies of meningioma cell cultures. As for microRNAs 181c and 130a, it was not possible to demonstrate a difference in expression in tissue or in plasma.
